# Biogenesis and Biological Functions of Extracellular Vesicles in Cellular and Organismal Communication With Microbes

**DOI:** 10.3389/fmicb.2022.817844

**Published:** 2022-02-18

**Authors:** Yuan Fang, Zhiwen Wang, Xili Liu, Brett M. Tyler

**Affiliations:** ^1^Department of Plant Pathology, College of Plant Protection, China Agricultural University, Beijing, China; ^2^College of Landscape and Ecological Engineering, Hebei University of Engineering, Handan, China; ^3^Department of Botany and Plant Pathology, Oregon State University, Corvallis, OR, United States

**Keywords:** extracellular vesicles (EVs), biogenesis, pathology, pathogen-plant interaction, cell to cell communication

## Abstract

Extracellular vesicles (EVs) represent a prominent mechanism of transport and interaction between cells, especially microbes. Increasing evidence indicates that EVs play a key role in the physiological and pathological processes of pathogens and other symbionts. Recent research has focused on the specific functions of these vesicles during pathogen-host interactions, including trans-kingdom delivery of small RNAs, proteins and metabolites. Much current research on the function of EVs is focused on immunity and the interactions of microbes with human cells, while the roles of EVs during plant-microbe interactions have recently emerged in importance. In this review, we summarize recent research on the biogenesis of these vesicles and their functions in biology and pathology. Many key questions remain unclear, including the full structural and functional diversity of EVs, the roles of EVs in communication among microbes within microbiomes, how specific cargoes are targeted to EVs, whether EVs are targeted to specific destinations, and the full scope of EVs’ transport of virulence effectors and of RNA and DNA molecules.

## Characteristics and Composition of Extracellular Vesicles

**FIGURE 1:**
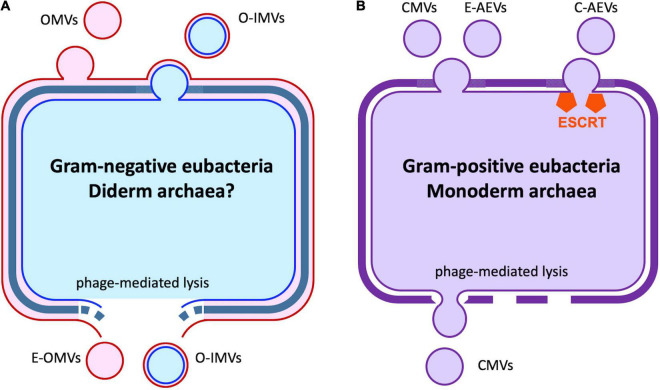
Extracellular vesicles produced by bacteria and archaea. **(A)** Gram-negative eubacteria release outer membrane vesicles (OMVs) by budding. A similar process results in the formation of outer-inner membrane vesicles (O-IMVs), with remodeling of the cell wall. Cell lysis induced by phage or environmental stress can also release O-IMVs and explosive OMVs (E-OMVs). It is unknown if similar processes occur in the small number of diderm archaeal species. **(B)** In gram-positive eubacteria and monoderm Euryarcheota, cytoplasmic membrane vesicles (CMVs) are released *via* cell membrane budding and cell wall re-modeling or as the result of cell lysis where the vesicles are extruded through gaps in the rigid cell wall. In the Crenarcheaota, CMVs are released *via* the action of the archaeal ESCRT machinery. See also Table 1.

**FIGURE 2:**
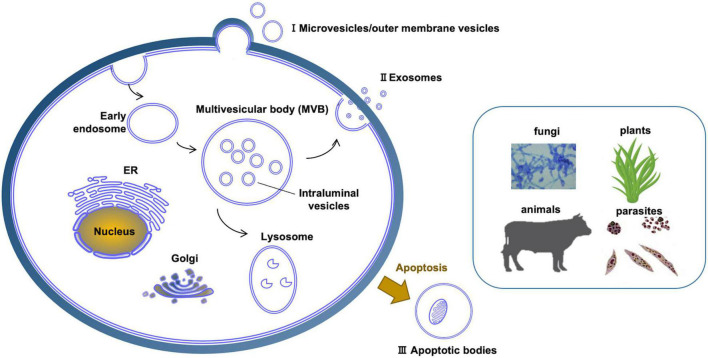
Different kinds of extracellular vesicles produced across diverse eukaryotic kingdoms. (I) Microvesicles are produced directly by pinching off of the plasma membrane. (II) Exosomes are produced through the intermediate structure of multivesicular bodies. (III) Apoptosis results in apoptotic bodies that constitute another type of extracellular vesicle. In plants and fungi, extracellular vesicles must cross a cell wall.

Extracellular vesicles (EVs) are a heterogeneous group of spheroid or cup-shaped membranous structures released by living cells that function in extracellular spaces. EVs have been observed in a wide diversity of prokaryotes and eukaryotes (Yoon et al., 2014; Woith et al., 2019; Díaz-Garrido et al., 2021a,b; Liu et al., 2021a). EVs could transfer a wide range of bioactive molecules including enzymes, sterols, phospholipids, polysaccharides, pigments, toxins and nucleic acids (Oliveira et al., 2013; Brown et al., 2015; Peres da Silva et al., 2015; Woith et al., 2019; Munhoz da Rocha et al., 2020). Studies in bacteria found that both Gram-negative and Gram-positive bacteria can produce EVs, usually called bacteria-derived EVs (BEVs). Gram-negative bacteria-produced BEVs can be divided into three types, namely outer membrane vesicles (OMVs), outer-inner membrane vesicles (O-IMVs), and explosive outer membrane vesicles (E-OMVs) (Table 1 and Figure 1A). Unlike Gram-negative bacteria, Gram-positive bacteria have a single cell membrane surrounded by a thick, rigid cell wall, and EVs produced by Gram-positive bacteria consist of cytoplasmic membrane vesicles (CMVs) (Díaz-Garrido et al., 2021a; Table 1 and Figure 1B). Archaeal EVs have been classified by differences in biogenesis and taxa of origin, crenarchaeotal AEVs (C-AEVs) produced *via* the archaeal ESCRT machinery, and euryarchaeotal AEVs (E-AEVs) produced *via* cell membrane blebbing (Liu et al., 2021a; Table 1 and Figure 1). Eukaryotic EVs have been divided into three classes: exosomes, microvesicles (MVs) and apoptotic cell-derived vesicles (Woith et al., 2019; Munhoz da Rocha et al., 2020; Cai et al., 2021; Figure 2 and Table 1). Exosomes are the smallest eukaryotic EVs with approximate dimensions ranging from 30 nm to 150 nm. Exosomes form intracellularly through inward budding of endosomal membranes resulting in the formation of intraluminal vesicles (ILVs) within multivesicular bodies (MVBs). Mature MVBs then fuse with the plasma membrane to release the ILVs as exosomes into the extracellular space. Microvesicles, sometimes called ectosomes, are formed directly by outward budding of the plasma membrane. They are generally larger than exosomes, and range in size from 100 to 1,000 nm. Apoptotic bodies are the largest EVs, with sizes usually ranging from 800 to 1,000 nm. They are formed from cells during programmed cell death (Joffe et al., 2016). These three classes of EVs have overlapping dimensions and components, and are similar in shape and density. Thus it remains difficult to cleanly isolate and distinguish the different classes of EVs. Furthermore, it remains unclear whether there are additional subtypes of each class of EVs, perhaps with distinctive contents, functions or destinations. For example, in plants at least three additional classes of EVs have been identified (Cai et al., 2021): low-density EVs lacking tetraspanins but containing the plant-specific syntaxin, PEN1; EVs derived from exocyst-positive organelles (EXPOs); and specialized secretory nanovesicles, called pollensomes, released by germinating pollen (Table 1). Thus, rapid and effective technologies to isolate and purify different classes and sub-types of EVs, perhaps by affinity methods targeting specific proteins or lipids (Cai et al., 2021), will be important to advance the study of the formation and function of EVs.

**TABLE 1:** Characteristics and biogenesis of different extracellular vesicles (EVs).

	EV type^a^	Size^a^	Origin^a^	References^a^
Archaeal EVs (AEVs)	Crenarchaeotal AEVs (C-AEVs)	90–230 nm	Archaeal ESCRT machinery	Liu et al., 2021a
	Euryarchaeotal AEVs (E-AEVs)	50–150 nm	Budding of the cell membrane	Liu et al., 2021a
Bacterial EVs (BEVs)	Outer membrane vesicles (OMVs)	20–300 nm	Blebbing of the outer membrane of gram negative bacteria	Díaz-Garrido et al., 2021a
	Outer-inner membrane vesicles (O-IMVs)	60–160 nm	Blebbing of the inner and outer membrane of gram negative bacteria	Pérez-Cruz et al., 2015
	Explosive outer membrane vesicles (E-OMVs)	110–800 nm	Phage-mediated cell lysis of gram negative bacteria	Turnbull et al., 2016; Toyofuku et al., 2017
	Cytoplasmic membrane vesicles (CMVs)	20–400 nm	Budding or extrusion of the cell membrane and release through cell wall pores or holes of gram-positive bacteria	Toyofuku et al., 2019; Briaud and Carroll, 2020
Traditional subtypes of eukaryotic EVs	Exosomes	30–150 nm	Released by multi-vesicular bodies fusing with plasma membrane	Joffe et al., 2016
	Microvesicles	100–1,000 nm	Outward budding of the plasma membrane	Joffe et al., 2016
	Apoptotic bodies	800–1,000 nm	Programmed cell death	Joffe et al., 2016
Additional subtypes of EVs in plants	Tetraspanin-positive EVs	Unclear^b^	Released by multi-vesicular bodies fusing with plasma membrane	Cai et al., 2018
	Penetration1-positive EVs	Unclear	Unclear	Rutter and Innes, 2017
	Exocyst-positive organelle-derived EVs	200–500 nm	Unclear	Wang et al., 2010
	Pollensomes	28–60 nm	Pollen-released secretory nanovesicles	Prado et al., 2014

*^a^Details in each column (from left to right) describe: the classes of EVs, the size range of EVs, the biogenesis mechanism of EVs, and the primary literature references.*

*^b^Published data do not provide a clear answer.*

## Multivesicular Body-Mediated Exosome Formation in Mammals and Plants

**FIGURE 3:**
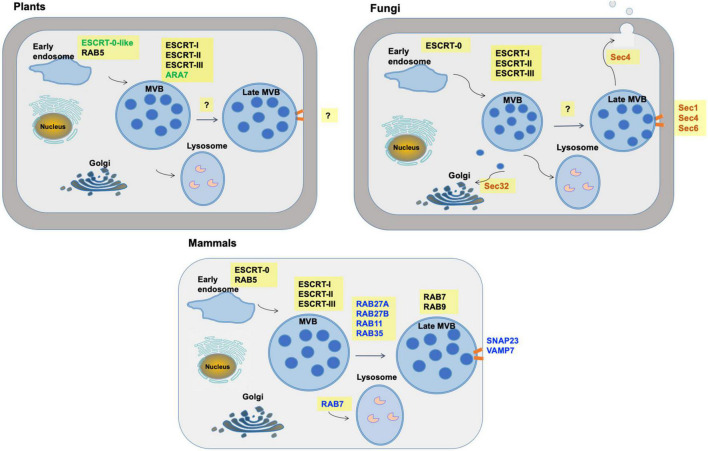
Multivesicular body pathways of exosome formation and release in mammals, plants and fungi. Commonalities, differences and unknowns are shown in the machinery by which exosomes are formed *via* multivesicular bodies (MVBs). Machinery which differs is indicated with colored text. Orange bars indicate fusion of MVBs with the plasma membrane. “?” indicates machinery that has not yet been well defined.

The mechanisms of exosome biogenesis have been extensively characterized (Figure 3), especially in mammalian cells, where at least four pathways of exosome biogenesis have been identified. They include pathways dependent on tetraspanins, metabolism of specific lipids, the Endosomal Sorting Complex Required for Transport (ESCRT) machinery, and pathways that require all three of these mechanisms (Colombo et al., 2014; Vietri et al., 2020).

Tetraspanins are a family of transmembrane proteins with four transmembrane domains and two extracellular domains. The larger extracellular domain, EC2, contains a stretch of highly conserved amino acids. There are 34 tetraspanins in mammals and 17 in the model plant *Arabidopsis thaliana*. Of these, five in mammals (CD9, CD63, CD37, CD81, and CD82) and two in *Arabidopsis* (TET8 and TET9) have been associated with exosomes (Cai et al., 2021).

**FIGURE 4:**
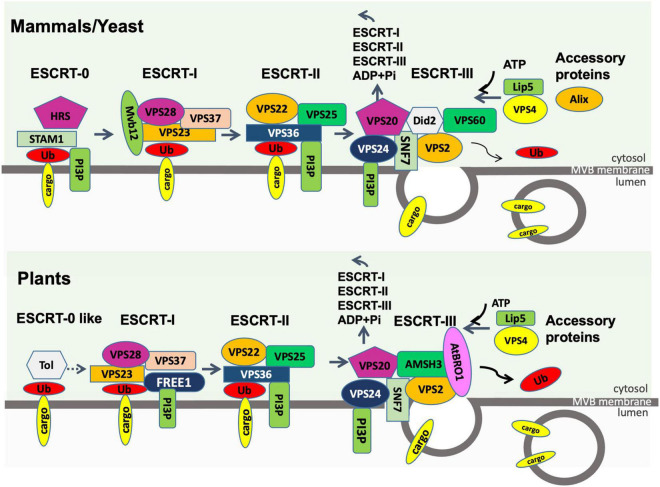
Endosomal sorting complex required for transport (ESCRT) machinery in plants compared to that in mammals and fungi. The process of loading ubiquitinated membrane cargoes into exosomes is shown. Ub, ubiquitin; PI3P, phosphatidylinositol-3-phosphate; ADP, adenosine diphosphate; Pi, phosphate. All other entities shown are protein components of the ESCRT machinery. The first step in the plant pathway is not well described.

The mammalian ESCRT machinery is composed of 4 protein complexes, namely ESCRT-0, –I, –II, and –III plus several accessory proteins, such as Alix, VPS4, and VTA-1 (Colombo et al., 2014; Figure 4). The ESCRT machinery is both important to deliver cargo into the MVB prior to exosome formation, as well as formation of the MVBs. ESCRT-0 consists of the protein STAM1 and the phosphatidylinositol-3-phosphate (PI3P)-binding protein HRS (hepatocyte growth factor-regulated tyrosine kinase substrate). PI3P-binding recruits ESCRT-0 to the endosomal membrane, where HRS recruits the ESCRT-I complex *via* its TSG101 subunit. ESCRT- II is then recruited and together with ESCRT-I act to deform the endosomal membrane inward to form buds. ESCRT- II or associated protein Alix then recruits ESCRT-III which is responsible for vesicle scission and formation of ILVs within the MVBs. Finally, the AAA-ATPase VPS4 mediates the dissociation of the ESCRT machinery from the membrane. The interactions between the ESCRT proteins and the soluble cargo proteins they are responsible for sorting into ILVs and exosomes are still not fully understood. ESCRT-0 carries 10 binding sites for polyubiquitinated proteins and therefore efficiently recruits ubiquitinated cargo proteins to the endosomal membrane during ILV formation (Vietri et al., 2020). ESCRT-I and ESCRT-II also include ubiquitin-binding subunits. Some evidence suggests that the chaperone HSC70 can bind to proteins with a KFERQ motif and also to phosphatidylserine on the outer membrane of the maturing MVBs, providing a ubiquitin-independent recruitment pathway (Sahu et al., 2011). Also, the ESCRT-II accessory protein Alix can bind some cargoes directly (Vietri et al., 2020).

After MVB maturation, fusion of MVBs with the plasma membrane is regulated by the RAB family of small GTPase proteins. The RAB family is composed of over 60 members and it is important in vesicle transport between multiple cellular compartments (Stenmark, 2009). Of these, RAB11, RAB27, and RAB35 play key roles in targeting of MVBs to the plasma membrane (Kowal et al., 2014; Blanc and Vidal, 2018). MVB fusion with the membrane resulting in the release of exosomes is mediated by SNARE (soluble *N*-ethylmaleimide-sensitive fusion attachment protein receptor) proteins, namely VAMP7 (vesicle-associated membrane protein 7) located on the MVBs and SNAP23 on the plasma membrane (D’Souza-Schorey and Schorey, 2018).

Homologs of most ESCRT proteins are found in plants (Figure 4), with the exception of ESCRT-0 proteins (Gao et al., 2017). The plant-specific ESCRT protein FREE1 (FYVE domain protein required for endosomal sorting 1) may replace the role of the ESCRT-0 complex. Like ESCRT-0, FREE1 could bind both PI3P and ubiquitinated proteins. Furthermore, FREE1 could bind directly to the ESCRT-I complex *via* its VPS23 subunit (Gao et al., 2014). Silencing FREE1 (Gao et al., 2014) or mutations in subunits of ESCRT-II (Wang H. J. et al., 2017), ESCRT-III (Shahriari et al., 2010; Cai et al., 2014; Yang et al., 2016), or ESCRT accessory proteins (Cai et al., 2014; Wang et al., 2015) in *A. thaliana* all affected normal formation of MVBs. A further difference from mammalian cells was that the ESCRT-III complex appeared more important to MVB formation than ESCRT-II in mammals (Gao et al., 2017). The mechanism of MVB targeting to the plasma membrane for release of exosomes is not well understood in plants. It has been hypothesized that, during plant infection, MVBs destined for vacuolar fusion are redirected to the site of infection to deliver exosomes containing defense molecules (Hansen and Nielsen, 2018). Tao et al. (2019) observed that the plasma membrane-localized ARM-E3 ubiquitin ligase, SAUL1, and its paralog AtPUB43, could mediate tethering of the MVBs to the plasma membrane during infection of *Nicotiana benthamiana* by *Phytophthora capsici.*

## Extracellular Vesicle Biogenesis and Release in Fungi

Fungal EVs were first isolated and characterized from culture supernatants of the human pathogen *Cryptococcus neoformans* (Rodrigues et al., 2007). Since then, related research has been reported in 11 additional fungal species (Rodrigues and Casadevall, 2018). EVs of *Cryptococcus gatti* effectuate long-distance coordination of virulence between fungal cells engulfed in different macrophages, while at the population level, secretion of EVs in *Candida albicans* is important for biofilm formation and antifungal resistance (Kwon et al., 2021). The mechanisms of biogenesis and release of fungal EVs are not as well elucidated as in mammals (Figure 3). Genetic studies have implicated at least three different pathways in the release of EVs in fungi, including the conventional post-Golgi secretory pathway, ESCRT-mediated release of exosomes *via* MVBs (Figure 4), and an unconventional secretory pathway involving Golgi reassembly stacking proteins (GRASP).

### Conventional Post-golgi Secretory Pathway

Several proteins related to components of this pathway have been knocked down to study the biogenesis mechanism of EV formation in *Saccharomyces cerevisiae* and *C. neoformans.* In *S. cerevisiae*, mutations in Sec1 and Sec4, which are small GTPases involved in membrane fusion and vesicle targeting, respectively, either altered EV composition or the kinetics of release, or both (Oliveira et al., 2010). In *S. cerevisiae* and *C. neoformans*, *Sec32* mutations affected EV composition; Sec32 is involved in trafficking from the ER to the Golgi (Oliveira et al., 2010). Furthermore, mutations in Sec6 reduced EV release by *C. neoformans*; Sec6 is a component of the exocyst which is responsible for delivery of post-Golgi exocytic vesicles to the plasma membrane prior to exocytic membrane fusion (Mei and Guo, 2018).

### Endosomal Sorting Complex Required for Transport-Mediated Release of Exosomes *via* Multivesicular Bodies

In line with the involvement of the ESCRT machinery in the release of EVs from metazoan cells, disruption of fungal genes encoding ESCRT subunits impacted the secretion and composition of EVs. In *S. cerevisiae*, mutations in the genes encoding the VPS2, VPS23, and VPS36 subunits affected EV abundance and proteomic profiles (Zhao et al., 2019) and similar results were observed in *C. albicans* (Zarnowski et al., 2018). In *C. neoformans*, mutations in the *VPS27* gene resulted in abnormal MVB and EV morphology (Park et al., 2020). However, in no cases could the mutations individually block the secretion of the vesicles, and indirect effects of the mutations on EV biogenesis could not be ruled out. These results are consistent with there being multiple alternative mechanisms for formation of EVs, and experiments with many combinations of mutations may be needed to dissect EV production in fungi.

### Unconventional Secretory Pathway Involving Golgi Reassembly Stacking Proteins

The principal function of GRASP proteins is to maintain the stacks of flat cisternal membranes that constitute the Golgi (Ahat et al., 2019), and to regulate autophagosome-lysosome fusion. However, in mammals, the GRASP55 protein (Ahat et al., 2019) and in yeast, the *GRH1*-encoded GRASP protein (Oliveira et al., 2010), have been implicated in unconventional secretion of several proteins lacking secretory leaders, including acyl-CoA binding protein (ACBP) (Ahat et al., 2019). Relevant to this review, Oliveira et al. (2010) observed that *grh1* mutants exhibited decreased release of EVs, though they could not distinguish between a direct role in EV biogenesis and indirect effects on the cellular distribution of sphingolipids which are an important component of EVs. GRASP was also shown to participate in EV-mediated export of mRNA in *C. neoformans* (Peres da Silva et al., 2018). In yeast, unconventional secretion of ACBP was shown to involve packaging of the protein into autophagosomes, fusion of the autophagosomes with early endosomes, maturation of the endosomes into MVBs, and fusion of the MVBs with the plasma membrane, likely releasing ACBP-containing EVs (Duran et al., 2010). However, it remains unclear if this is a general pathway for release of EVs, or one specific to ACBP.

In addition to transport across the plasma membrane, EVs must traverse the cell wall in the case of organisms that have them, such as plants, bacteria, fungi and oomycetes (Woith et al., 2019). The ability of liposomes containing amphotericin B to enter fungal cells (Walker et al., 2018) and the evidence for cross-kingdom communication *via* EVs (Cai et al., 2018) strongly implies that EVs can also traverse cell walls from the outside inward. Despite extensive observations in *C. neoformans* (e.g., Wolf et al., 2014), the mechanisms of EV cell wall transit remains unclear. Current hypotheses include: the presence of guide channels, the actions of cell-wall-remodeling enzymes, and intrinsic visco-elasticity of cell walls (Rizzo et al., 2020). It also remains unclear if the directional transit of EVs requires specific transport mechanisms as is observed for the trafficking of intracellular vesicles.

## Production of Extracellular Vesicles by Bacteria and Archaea

The release of BEVs by bacteria was first observed in *Vibrio cholerae* in 1967 (Chatterjee and Das, 1967), where they were described as round membranous structures produced by budding and detachment from the bacterial outer membrane. The mechanisms underlying BEV biogenesis remain poorly understood. Processes associated with BEV release include cell wall turnover; physical, salt, or antibiotic-induced stress; lipopolysaccharide and phospholipid remodeling of the outer membrane; and the action of quorum-sensing signal molecules (Díaz-Garrido et al., 2021a). OMVs have been characterized as containing cell wall components, lipopolysaccharides, enzymes and other proteins, and secondary metabolites, as well as DNA molecules and an array of RNA molecules including tRNAs, mRNAs, non-coding RNAs, and fragments of rRNAs (Woith et al., 2019; Munhoz da Rocha et al., 2020; Cai et al., 2021; Díaz-Garrido et al., 2021a). Gram-negative bacteria also produce “outer-inner membrane vesicles” (O-IMVs), likely by similar mechanisms as OMVs. O-IMVs exhibit both an outer and an inner membrane, and enclose both periplasmic and cytoplasmic contents, including DNA (Pérez-Cruz et al., 2015). In gram-positive bacteria, genetic analysis suggests that formation of BEVs is a normal physiological process; vesicles with cytoplasmic contents (CMVs) are pinched off from the cell membrane *via* lipid-remodeling, and then released through the cell wall *via* remodeling proteins that include autolysins and penicillin-binding proteins (Briaud and Carroll, 2020). Phage-mediated lysis can also produce “explosive OMVs” (E-OMVs) in gram-negative bacteria (Turnbull et al., 2016). In gram-positive bacteria, a similar process can result in CMVs being extruded through gaps in the cell wall (Toyofuku et al., 2017).

Archaea also produce EVs (AEVs). The majority of archaea have a single membrane and a flexible external cell wall-like structure, though a minority of species resemble gram-negative bacteria in having an outer membrane (Liu et al., 2021a). The mechanisms of AEV production differ markedly across phyla. Several archaea phyla encode orthologs of the eukaryotic ESCRT machinery, including the Crenarchaeota. In the Crenarchaeota, proteomic and genetic analysis, especially in *Sulfolobus islandicus* (Liu et al., 2021b), has demonstrated the importance of the ESCRT machinery in C-AEV production (Liu et al., 2021a). The Euryarchaeota, however, lack ESCRT homologs and E-AEV production appears to occurs by budding from the cell membrane *via* an unknown mechanism (Liu et al., 2021a). Like CMVs, AEVs enclose diverse cytoplasmic contents. However, both C-AEVs and E-AEVs appear to have a particular role in the encapsulation, stabilization, and cell-to-cell transmission of DNA fragments, including chromosomal fragments and plasmid molecules (Liu et al., 2021a), a process termed vesiduction (Erdmann et al., 2017).

## Biological Functions of Extracellular Vesicles in Host-Microbe Interactions

**FIGURE 5:**
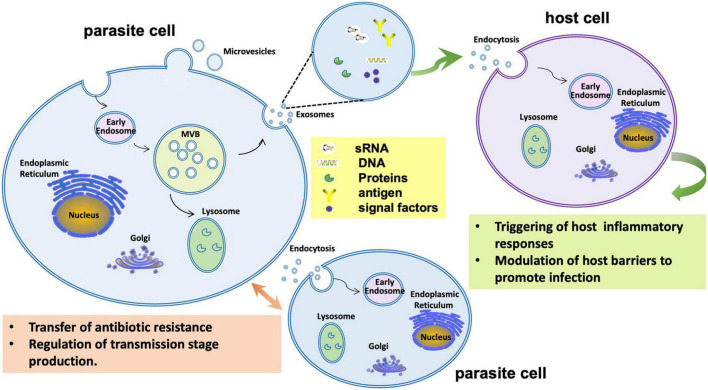
Extracellular vesicle functions during infection by the malaria parasite *Plasmodium falciparum*. During infection *P. falciparum* EVs may trigger host inflammatory responses or manipulate gene expression to reduce host barriers to infection. *P. falciparum* EVs may also mediate communication among parasite cells to regulate transmission stage production or even transfer antibiotic resistance.

**FIGURE 6:**
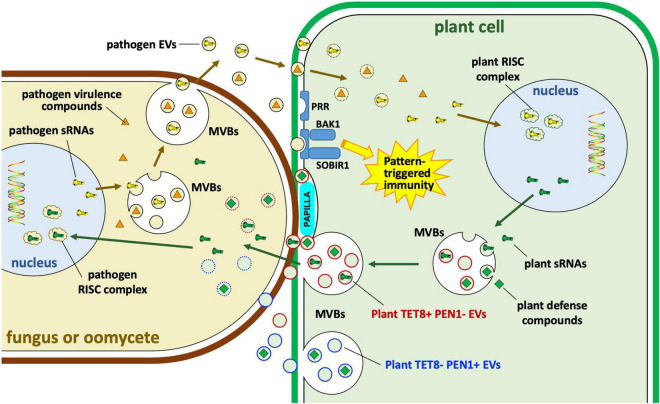
Extracellular vesicle roles in interactions of plants with fungi and oomycetes. Plants and their eukaryotic pathogens exchange EVs during infection. Plant EVs carrying Tetraspanin8 (Tet8) but lacking syntaxin PEN1 (TET8+ PEN1– EVs) carry small RNAs (sRNAs) and other defense compounds including small molecules, enzymes and other proteins. The specific role of plant TET8-negative PEN1-positive (TET– PEN1+) EVs remains unclear. Some of those molecules may enter the pathogen cytoplasm or the nucleus where sRNAs may bind to pathogen RNA-induced silencing complexes (RISC); some enzymes such as callose synthase may be targeted to papillae to strengthen cell wall defenses against pathogen invasion. Pathogen EVs may carry sRNAs as well as other virulence compounds, possibly including some effectors. sRNAs may target plant RISC complexes. MVBs, multivesicular bodies; PRR, pattern recognition receptor; BAK1, BRASSINOSTEROID INSENSITIVE 1-associated receptor kinase 1; SOBIR1, suppressor of BRASSINOSTEROID INSENSITIVE 1 (BRI1)-associated kinase (BAK1)-interacting receptor kinase 1.

Multi-cellular hosts such as animals and plants host rich communities of microbes (microbiomes). These communities are typically dominated by eubacteria, but also may include archaea, fungi, oomycetes, protozoa, algae, viruses, and microfauna. The relationships among these organisms and with their hosts can range across all forms of symbiosis from parasitism, competition and commensalism to mutualism (Tyler, 2009). EVs are rapidly emerging as key players in the communications and physiological interactions among these microbes and their hosts (Woith et al., 2019; Caruana and Walper, 2020; Munhoz da Rocha et al., 2020; Cai et al., 2021; Díaz-Garrido et al., 2021a,b; Fucarino et al., 2021; Qiao et al., 2021; Figures 5, 6 and Table 2). These roles can include nutrition, virulence, predation, host defense, immuno-regulation, developmental signaling and environmental modification. While much work has focused on pathogenic microbes, there is also accumulating evidence that symbiotic microbes may also employ EVs to modulate host interactions (Roth et al., 2019; Rizzo et al., 2020; Cai et al., 2021; Díaz-Garrido et al., 2021a,b). In particular, EVs have emerged as a mechanism for the transport of RNA molecules from the microbe to the host in order to modulate host metabolism and immune responses (Munhoz da Rocha et al., 2020; Cai et al., 2021; Qiao et al., 2021). Likewise, the delivery of host RNA molecules to microbes has emerged as an important function of host EVs (Munhoz da Rocha et al., 2020; Cai et al., 2021; Qiao et al., 2021).

**TABLE 2:** Virulence factors delivered by EVs and function during pathogen infection.

Microbe	Virulence factors	Function	References
**Bacteria**			
*Pseudomonas aeruginosa*	Alkaline phosphatase, hemolytic phospholipase C, toxin Cif	Trigger pathological immune responses	Bomberger et al., 2009
*Escherichia coli*	Toxin V, EHEC- hemolysin	Trigger pathological immune responses	Bielaszewska et al., 2017
*Xyllela fastidiosa*	Lipases, esterases, proteases, porins, pectin lyase-like protein, and signaling factors	Unclear	Feitosa-Junior et al., 2019
*Bradyrhizobium japonicum*	Small RNAs	Unclear	Ren et al., 2019
**Fungi**			
*Candida albicans*	Agglutinins, lysophospholipases, and secreted aspartic proteases		Konecňá et al., 2019
*Cryptococcus gatti*	Unclear	Promote infection of pathogen in host	Bielska et al., 2018
*Sporothrix brasiliensis*	Unclear	Promote infection pathogen in host	Ikeda et al., 2018
*Beauveria bassiana*	Small RNAs, mRNAs	Modulate host immune responses	Cui et al., 2019
*Botrytis cinerea*	Small RNAs, mRNAs	Modulate host immune responses	Weiberg et al., 2013
*Verticillium dahliae*	Small RNAs, mRNAs	Modulate host immune responses	Wang et al., 2016
*Puccinia striiformis* f. sp. *tritici*	Small RNAs, mRNAs	Modulate host immune responses	Wang M. et al., 2017
*Ustilago. maydis*	Small RNAs, mRNAs	Modulate host immune responses	Kwon et al., 2021
**Oomycete**			
*Hyaloperonospora arabidopsidis*	Small RNAs, mRNAs	Modulate host immune responses	Dunker et al., 2020
**Nematode**			
*Heligmosomoides polygyrus*	microRNAs, Y RNAs, Argonaute protein	Suppress innate immune responses in mice	Buck et al., 2014
Apicomplexan parasite			
*Plasmodium falciparum*	miRNAs	Trigger host systemic inflammatory responses and promote infection by pathogen in host	Mantel et al., 2016

### Extracellular Vesicle Function in Plant and Animal Pathogens and Symbionts

Bacteria-derived extracellular vesicles produced by Gram-negative bacteria have been implicated in many aspects of bacterial pathogenicity and proliferation, including nutrient acquisition, stress responses, virulence factor delivery, biofilm formation, and development of antibiotic resistance (Kuehn and Kesty, 2005; Schwechheimer and Kuehn, 2015; Joffe et al., 2016; Toyofuku et al., 2019; Woith et al., 2019; Díaz-Garrido et al., 2021a). Virulence factors delivered by BEVs include alkaline phosphatase, hemolytic phospholipase C and Cif toxin in the case of *Pseudomonas aeruginosa* (Bomberger et al., 2009) and Shiga toxin 2a, cytolethal distending toxin V and EHEC-hemolysin in the case of enterohemorrhagic *Escherichia coli* (Bielaszewska et al., 2017). After being internalized by host cells, BEVs can release these virulence cargos and cause cell death (Bielaszewska et al., 2017). Mammalian host responses to BEVs can also trigger pathological immune responses, such as sepsis (Park et al., 2018). Although BEVs have been reported in bacterial plant pathogens, there is little information yet about their role in pathogenesis. One analysis of BEV-enriched fractions from the grape and citrus pathogen *Xyllela fastidiosa* revealed the presence of virulence factors including lipases, esterases, proteases, porins, and a pectin lyase-like protein, as well as diffusible signaling factors, suggesting that BEVs might have a role in delivering these factors (Feitosa-Junior et al., 2019). The identification of bacterial small RNAs as host-signaling molecules during nodulation of soybean by *Bradyrhizobium japonicum* (Ren et al., 2019) also suggests the involvement of BEVs as potential carriers of those RNAs.

The contributions of EVs to microbiome-host interactions have been studied in particular detail in the context of the bacterial members of mammalian microbiomes (Ñahui Palomino et al., 2021), especially those of the mammalian gut (reviewed in detail by Díaz-Garrido et al., 2021a,b) and airways (reviewed by Fucarino et al., 2021). In the gut, a dense inner layer of mucus separates the host epithelial cells from the microbial community, while a looser outer layer of mucus serves as a matrix that is colonized by the microbes. While these mucus layers restrict the movement of microbial cells including bacteria, BEVs can readily pass these barriers, physiologically connecting the cells with each other and with the host. One role played by the BEVs is a dietary one. BEV cargoes are rich in enzymes such as glycosidases, sulfatases, proteases, and inositol phosphatases that can break down host-indigestible dietary glycans and also host mucins, releasing nutrients such as carbohydrates, short chain fatty acids, and phosphates to both host and microbes. BEVs are also important effectors of the role of the gut microbiome in stimulating the maintenance of the epithelial barrier and providing a healthy ongoing priming of the innate immune system (Díaz-Garrido et al., 2021a). BEVs can also cross the epithelial layer into the body, likely *via* paracellular diffusion, transcytosis and/or *via* phagocytotic cells in the mucosal epithelium (Jones et al., 2020; Díaz-Garrido et al., 2021a), with physiological effects that are still being explored.

In mammalian airways, as in the gut, host EVs play critical roles in maintaining a healthy balance of immunological activities within the mucosa and its associated mucus layer (Fucarino et al., 2021). This balance can be disrupted by external factors such as allergens and tobacco smoke, resulting in asthma and chronic pulmonary obstructive disease (COPD). These disruptions can result in microbial dysbioses that include release of BEVs rich in pro-inflammatory signals, creating a positive feedback loop that exacerbates disease. One well-studied example is the interaction between airway epithelial cells and *Pseudomonas aeruginosa*, a gram-negative bacterium that causes dangerous infections in patients with COPD and cystic fibrosis. *P. aeruginosa* BEVs deliver a 23-nucleotide tRNA fragment (sRNA-52320) into epithelial cells that suppresses cytokine secretion and neutrophil infiltration, resulting in weakened immune responses (Koeppen et al., 2016).

While fungi and other eukaryotic microbes have been well documented to produce EVs, direct evidence for EVs’ role in the virulence of such pathogens is still emerging. In the human pathogen *C. albicans*, EVs produced *in vitro* were shown to contain 34 proteins associated with virulence including agglutinins, lysophospholipases, and secreted aspartic proteases (Konecňá et al., 2019). In the gastrointestinal nematode *Heligmosomoides polygyrus*, nematode EVs were shown to carry microRNAs, Y RNAs and an Argonaute protein, and could suppress innate immune responses in mice (Buck et al., 2014). In the human pathogens *C. gatti* (Bielska et al., 2018) and *Sporothrix brasiliensis* (Ikeda et al., 2018), EVs were shown to carry infection-promoting factors, though the factors were not identified. In the malaria parasite, *Plasmodium falciparum*, EVs derived from parasite-infected red blood cells (IRBCs) could trigger host systemic inflammatory responses and could contribute to the pathology of malaria infection by altering the host barrier properties of endothelial cells (Mantel et al., 2016).

An important role for EVs is also implied by data showing that small RNAs from fungi and oomycetes interact with the RISC machinery of hosts to modulate host immune responses (Figure 6). Examples include the entomopathogenic fungus *Beauveria bassiana* (Cui et al., 2019), the fungal plant pathogens *Botrytis cinerea* (Weiberg et al., 2013), *Verticillium dahliae* (Wang et al., 2016), *Puccinia striiformis* f. sp. *tritici* (Wang M. et al., 2017), and *Ustilago maydis* (Kwon et al., 2021), and the oomycete plant pathogen *Hyaloperonospora arabidopsidis* (Dunker et al., 2020). In these cases, EVs are hypothesized to carry both sRNAs and mRNAs to the host. In malaria, over 20 human miRNAs were identified in EVs that were released from IRBCs. Among them, miR-451a could modify the permeability of host endothelial cells by silencing target genes within those cells (Mantel et al., 2016; Figure 5).

Microbial pathogens of plants and animals produce effector proteins that modify the physiology and morphology of host tissues to promote infection (Torto-Alalibo et al., 2010). Many classes of effector proteins are delivered into the cytoplasm of host cells (Torto-Alalibo et al., 2010). While effector delivery pathways have been well characterized in bacteria (Tseng et al., 2009), the entry of effector proteins from fungal and oomycete pathogens is still poorly understood (Ellis et al., 2006; Khang et al., 2010; Kale and Tyler, 2011; Jiang and Tyler, 2012; Presti and Kahmann, 2017). In principle, EVs offer an attractive mechanism for effector delivery from fungi and oomycetes into the cytoplasm of host cells. Some evidence suggests that effectors targeted to the host cytoplasm are secreted *via* distinct pathways (Giraldo et al., 2013; Wang S. et al., 2017). Furthermore, some oomycete effectors appear to reach the host cytoplasm *via* non-conventional secretion pathways (Liu et al., 2014). However, clear evidence is currently lacking that would support the role of EVs in effector delivery.

### Extracellular Vesicle Functions in Host Disease and Immune Responses

Extracellular vesicles have emerged as major players in host responses to microbes. Host EVs can contribute to combatting microbial infection either by targeting pathogen cells directly, or by participating in the regulation of immune responses (Chen et al., 2019; Rybak and Robatzek, 2019; Macia et al., 2020; Cai et al., 2021; Figures 5, 6). At the same time, host responses triggered by microbial EVs may contribute to immunity but also may lead to disease symptoms (Chen et al., 2019; Rybak and Robatzek, 2019; Macia et al., 2020; Cai et al., 2021; Qiao et al., 2021). Common themes that have emerged across animal and plant interactions include recognition of microbe-associated molecular patterns (MAMPs) carried by microbial EVs by pattern recognition receptors (PRRs), resulting in the activation of innate immune responses *via* mitogen-activated protein kinase (MAPK) pathways (Zhou et al., 2020; Cai et al., 2021; Díaz-Garrido et al., 2021a,b). Some differences include the extensive role of mammalian EVs in modulating immune responses (Díaz-Garrido et al., 2021a,b), especially in the gut, while in plants, an important role of EVs is as carriers of anti-microbial RNA molecules (Cai et al., 2018, 2021). However, these differences may fade as investigations of these systems progress.

### Roles in Animal Disease and Immune Responses

During mammalian immune responses, host EVs have been documented to play a wide range of regulatory roles, including antigen presentation, immune homeostasis *via* pro- or anti-inflammatory mediators, and transfer of damage-associated molecular patterns (DAMPs), membrane receptors, enzymes, mRNAs and non-coding RNAs. EVs may also modulate the complement and coagulation systems (Chen et al., 2019; Macia et al., 2020). Mammalian immune systems also respond to EVs carrying pathogen-associated molecular patterns (PAMPs) that are recognized by pattern recognition receptors (PRRs), resulting in inflammasome activation, and activation of cells of the innate immune system. In some cases, host pro-inflammatory responses can become pathological, producing tissue damage and sepsis (Chen et al., 2019; Macia et al., 2020). For example, host microvesicles are greatly elevated in the blood of cerebral malaria patients, and were subsequently shown in a mouse model to be the principal effectors of neurovascular pathology, especially microvesicles derived from *Plasmodium*-infected red blood cells (Combes et al., 2005; El-Assaad et al., 2014; Macia et al., 2020). Immune responses may also be directed against microbial EVs. For example, binding of antibodies could modulate both the composition and the production of EVs by the fungal pathogen *Histoplasma capsulatum*, reducing its virulence in mice (Baltazar et al., 2018).

The roles of host EVs in maintaining healthy mucosal surfaces in the presence of abundant microbial populations have been extensively studied in the mammalian gut (reviewed in Díaz-Garrido et al., 2021b). In the gut, EVs mediate extensive communications among epithelial cells, phagocytic cells, dendritic cells, mast cells and mesenchymal stem cells. The cargos of these EVs include not only cytokines and other regulatory proteins but also a wide spectrum of miRNAs involved in immuno-modulation (Díaz-Garrido et al., 2021b). The functions of those cargoes include support of epithelial barrier integrity, tissue repair and wound healing, intestinal immune responses, pathogen control, and microbiota modulation (Díaz-Garrido et al., 2021b). Furthermore, study of BEVs produced by probiotic bacteria, such as *E. coli* Nissle 1917 (EcN) (Cañas et al., 2016) and kefir *Lactobacillus* strains (Seo et al., 2018) demonstrated that BEVs produced by these strains could reduce the increased expression of pro-inflammatory cytokines and down-regulate enzymes associated with injury and inflammation (Díaz-Garrido et al., 2021b). Associations of host EVs with airway pathologies present in asthma and COPD have also been extensively documented (reviewed in Fucarino et al., 2021). While EVs may be expected to play similar key roles in the interaction of plants with their microbiomes, this research area is still in its infancy (Motaung and Steenkamp, 2021).

### Roles in Plant Disease and Immune Responses

Extracellular vesicles from plants have been demonstrated to directly participate in anti-microbial functions (Figure 6). EV production by plants is stimulated by infection as well as by activators of immune signaling such as salicylic acid (Rybak and Robatzek, 2019). One important role of EVs in plant defense is to target the deposition of cell-wall-strengthening callose to sites of attempted pathogen invasion, which is part of the polarized defense response. The polarized defense response has been observed in diverse plant species, including barley, bean and *Arabidopsis*, protecting against diverse fungal pathogens (Rybak and Robatzek, 2019). EVs also target chemical defenses to sites of pathogen attack. In *Arabidopsis*, products of indole glucosinolate metabolism form important anti-microbial defenses against fungi and insects. Glucosinolate transporters and the activating enzyme, myrosinase, are associated with *Arabidopsis* EVs during infection (Rybak and Robatzek, 2019). In sunflower, EVs from seedlings could deliver defense proteins to ascospores of *Sclerotinia sclerotiorum* and could inhibit their germination and growth (Regente et al., 2017).

In plants, EVs have emerged as carriers of host small RNAs that target pathogen genes (Figure 6). TET8-positive exosomes from *Arabidopsis* were found to be enriched in a selective set of miRNAs and small interfering RNAs (siRNAs), including phased secondary siRNAs (phasiRNAs). Those exosomes could readily be taken up by the fungal pathogen *Botrytis cinerea*. Furthermore, specific sRNAs such as TAS1c-siR483 and TAS2-siR453 could regulate the expression of fungal pathogenicity genes *Bc-VPS51*, *Bc-DCTN1* and *Bc-SAC1* (Cai et al., 2018). *Arabidopsis* also produced phasi-RNAs capable of silencing genes in the pathogen *P. capsici* (Hou et al., 2019). *Arabidopsis tet8, tet9* double knock-out mutants were substantially more susceptible to *B. cinerea*, and *B. cinerea* hyphae from infected *tet8, tet9* plants contained substantially fewer plant sRNAs (Cai et al., 2018).

The ability of EVs to deliver sRNAs from plants to pathogens likely underlies the phenomenon of host-induced gene silencing, in which silencing RNAs produced from transgenes or endogenous genes can effectively target pathogen genes to reduce disease (Baulcombe, 2015; Koch and Wassenegger, 2021; Qiao et al., 2021). In a recent study, EVs isolated from *Arabidopsis* leaves contained siRNAs derived from a transgene-encoded dsRNA targeting three *CYP51* genes of the fungal pathogen *Fusarium graminearum*, conferring increased resistance against this pathogen. Disruption of the ESCRT III complex, which is vital to the EV formation in plants, weakened the resistance of these transgenic plants against *F. graminearum* (Koch et al., 2020).

The plant innate immune system also responds to the presence of microbial EVs (Bahar et al., 2016; Rybak and Robatzek, 2019; McMillan et al., 2021; Figure 6). For example, outer membrane-derived EVs (OMVs) from *Xanthomonas campestris* pv. *campestris* could induce a reactive oxygen species (ROS) burst and trigger defense gene expression in *Arabidopsis* (Bahar et al., 2016). The EVs could be perceived by the plant coreceptor brassinosteroid insensitive 1 associated kinase (BAK1) and suppressor of BAK1 interacting receptor-like kinase 1 (SOBIR1) to induce PAMP-triggered immunity (PTI) (Bahar et al., 2016). Similarly, exposure to OMVs from *Pseudomonas syringae* and *Pseudomonas fluorescens* could protect *Arabidopsis* and tomato plants from infection by *P. syringae* or by oomycetes (McMillan et al., 2021).

## Function of Extracellular Vesicles in Cell-to-Cell Communication

Extracellular vesicles play an important role not only in microbe-host interactions but are also important to intercellular communication among microbes, either of the same or different species, and among cells of higher organisms (Caruana and Walper, 2020; Munhoz da Rocha et al., 2020; Díaz-Garrido et al., 2021b).

For instance, within mammals, EVs shed from stem cells contained mRNA and miRNAs which could alter the phenotype of other stem cells (Ratajczak et al., 2006). As another example, tumor cell-derived EVs contained abundant soluble proteins, signal factors and chemokine receptors, that could promote tumor invasion and influence tumor progression (Turturici et al., 2014). Within the mammalian gut, host EVs play a key role, together with microbial EVs, in maintaining a balanced immunological and mucosal environment that controls microbiota populations at a beneficial level, while avoiding pathological levels of inflammation (Díaz-Garrido et al., 2021b).

The role of EVs in microbe-microbe communication within complex microbial communities (microbiomes) has been examined in detail in the context of the human gut, especially the role of BEVs (Caruana and Walper, 2020). The majority of beneficial community functions mediated by BEVs involve “common good” functions such as provision of BEVs carrying carbohydrate degradation enzymes, iron acquisition proteins, antibiotic or antimicrobial resistance molecules (enzymes such as ß-lactamases or catalases, or excess antibiotic target proteins or lipids), and biofilm matrix components such as DNA (Caruana and Walper, 2020; Munhoz da Rocha et al., 2020). For example, in the bacterium, *E. coli*, BEVs released from β-lactam antibiotic-resistant strains contained abundant antibiotic-resistance proteins including Blc, OmpC, OmpF, and OmpW. Cells of antibiotic-susceptible strains could incorporate these resistance proteins from BEVs, protecting themselves from the antibiotic inhibition (Kim et al., 2018). The transmission of developmental signals directly from cell to cell by BEVs and AEVs includes the transfer of DNA molecules and quorum sensing signals. For example, the *P. aeruginosa* quorum sensing molecule PQS (*Pseudomonas* Quinolone Signal) is highly hydrophobic and primarily exported on the surface of BEVs (Mashburn and Whiteley, 2005). Similarly, the hydrophobic quorum sensing molecules of *Paracoccus denitrificans* and *Vibrio harveyi* are packaged into BEVs (Toyofuku et al., 2017; Brameyer et al., 2018). On the other hand, developmental signaling by transfer of protein or RNA molecules among eubacteria has yet to be demonstrated.

While the mechanisms by which bacteria, especially gram-negatives, could internalize incoming EVs remain speculative, the mechanisms for eukaryotes to internalize EVs have been extensively studied in mammalian cells (Caruana and Walper, 2020). Five different endocytic pathways have been identified by which EVs can be taken into non-phagocytic host cells: macropinocytosis, clathrin-mediated endocytosis, caveolin-mediated endocytosis, lipid raft-mediated endocytosis, and direct membrane fusion (Caruana and Walper, 2020). By extrapolation therefore, eukaryotic microbes are expected to be efficient recipients of EVs, as demonstrated in plant-fungal interactions (Cai et al., 2018, 2021). For example, in the human fungal pathogen *C. gattii*, EVs could induce pathogen proliferation and regulate the virulence phenotype of the recipient pathogen cells over a long distance (Bielska et al., 2018). EVs released from a highly virulent strain of *C. gattii* could be phagocytosed by host macrophages and there stimulate the growth rate of a less virulent strain and enhance its survival. The components transported in EVs were important to induce fungi proliferation, and only EVs from the virulent strain had this capability. However, the exact nature of the stimulatory molecules and the mechanism of stimulation were unknown (Bielska et al., 2018). Likewise, during infection by the malaria parasite *Plasmodium falciparum*, EVs purified from IRBCs could transfer drug-resistance plasmids into drug sensitive strains and increase survival of the parasite under drug pressure (Regev-Rudzki et al., 2013; Figure 5). In addition, IRBC-derived EVs could regulate the transmission stage production of parasites *in vivo* (Mantel et al., 2013; Figure 5). The EVs could promote gametocyte formation in the parasite population; gametocytes are the form of the parasite transmitted to the mosquito vector. It was proposed that EVs released from IRBCs serve as a sensor for parasite density, signaling a switch to increased gametocyte formation (Mantel et al., 2013).

Complex microbial communities also are common in non-biotic environments such soils, sediments, rocks, and human-built structures, especially where water is abundant. These communities commonly form biofilms that serve to protect and structure the communities, and concentrate nutrients, metabolites and signal molecules. These biofilms likely are rich in microbial EVs. However, this topic is barely explored, except in the case of Archaea, which dominate environments of extreme temperatures, salinity and/or acidity. Two specific functions have been associated with AEVs, namely the dissemination of toxins that target competitors, and the transmission of genomic and plasmid DNA molecules. *Sulfolobus* species produce a family of AEV-associated anti-microbial proteins, called sulfolobicins, that inhibit the growth of related *Sulfolobus* species (Ellen et al., 2011). The encapsulation of DNA by EVs is found in multiple phyla including the Crenarchaeota and the Euryarcheaota. *Thermococcus onnurineus* EVs always contain random ∼14 kb fragments of DNA that represent most of the genome, suggesting that these AEVs mediate genetic exchange within the same or related species (Choi et al., 2015). AEVs capable of transmitting specific plasmids have also been described in *Thermococcus kodakarensis, Thermococcus nautili, Sulfolobus* species and *Halorubrum* species (Liu et al., 2021a,b). In addition to these specific functions, AEVs have been observed to carry a wide diversity of proteins, including diverse proteases and nucleases (Liu et al., 2021b) and could support heterotrophic growth of *S. islandicus* in minimal medium, implying roles in nutrition (Liu et al., 2021a).

It is likely that EVs play key roles in cell-cell communication relevant to a very wide range of biological activities, beyond the few examples given above. There is growing evidence, for example, that BEVs can transit gut mucosal barriers and reach intestinal immune cells, the blood, and distal organs such as the brain, with measurable physiological effects (Díaz-Garrido et al., 2021a), opening further frontiers in the interaction between microbes (from all kingdoms) and multicellular animals. It is likely also that EVs play similarly important roles in cell-cell communication in plants and in plant-associated microbes, though studies on this topic are currently lacking. The role of EVs in communication among microbes that constitute microbial communities (microbiomes), especially the exchange of developmental signals, remains largely unexplored.

## Conclusion and Future Directions

Extracellular vesicles have emerged as an exciting and long under-appreciated component of cells. EVs extend the sphere of influence of cells well beyond what can be achieved by conventional secretory mechanisms. The importance of EVs is underlined by the finding that they are produced by all forms of living organisms, including numerous kingdoms of microbes. Because of their ability to transport cell contents, including RNA and DNA, from one organism to another, they have emerged as key players in microbial interactions with plants and animals, as well as in diseases such as cancer. As such they have also emerged as useful biomarkers for diagnosis of some clinical diseases and are being modified for delivery of vaccines and other therapies (Robbins and Morelli, 2014). Compared with the research into mammalian, parasite and bacterial EVs, research into the functions of fungal and oomycete EVs is still in its infancy, especially in the context of plant interactions, and many questions need to be addressed.

A major challenge for EV research is the ability to discern and purify different sub-types of EVs. While progress has been made on separating EVs by size and density, and in some cases immunologically (Rybak and Robatzek, 2019), a much more complete set of biomarkers is needed to accurately determine the origins, contents, functions and targeting of diverse EVs. This information is also needed to fully realize the potential of synthetic EVs as therapeutic agents (Huyan et al., 2020). Key questions remain around the contents of EVs, including whether and how specific cargoes are loaded into EVs. Cargoes of special interest include virulence effector proteins produced by fungal and oomycete pathogens of plants and animals. How these effectors are delivered into host cells remains poorly understood, and EVs stand out as a likely delivery mechanism, both for conventionally and unconventionally secreted effectors. Another cargo of major interest is RNA molecules. Strong evidence has emerged, especially from plant systems, that hosts and pathogens can use EVs to convey specific RNA molecules to each other to modify the physiology, immunity and virulence of their antagonist (Cai et al., 2018). However, the full scope of this mechanism remains to be explored. For example, is EV delivery confined to small RNAs or are full length mRNAs or non-coding RNAs conveyed by this mechanism? Also, what counter-measures might the antagonists have evolved against each other’s EVs (Qiao et al., 2013, 2021)? Another interesting question is whether EVs may play a role in horizontal gene transfer between species or even between kingdoms. Finally, the roles of EVs in communication within communities of microbes (microbiomes) remain largely unexplored.

## Author Contributions

YF and BT wrote the manuscript with contributions from all authors. YF, ZW, XL, and BT conceived the manuscript, read, and agreed to the published version of the manuscript.

## Conflict of Interest

The authors declare that the research was conducted in the absence of any commercial or financial relationships that could be construed as a potential conflict of interest.

## Publisher’s Note

All claims expressed in this article are solely those of the authors and do not necessarily represent those of their affiliated organizations, or those of the publisher, the editors and the reviewers. Any product that may be evaluated in this article, or claim that may be made by its manufacturer, is not guaranteed or endorsed by the publisher.
